# Peripapillary hyper-reflective ovoid mass-like structure and dome-shaped maculopathy: A case report

**DOI:** 10.1097/MD.0000000000028652

**Published:** 2022-01-21

**Authors:** Wei Zhang, Da-Guang Bi, Xiao-Yan Peng, Wei Gu

**Affiliations:** a Department of the Ophthalmology, Beijing Aier Intech Eye Hospital, Beijing, China; b Department of the Ophthalmology, Beijing Tongren Eye Hospital, Capital Medical University, Beijing, China.

**Keywords:** dome-shaped maculopathy, high myopic, peripapillary hyper-reflective ovoid mass-like structure

## Abstract

**Rationale:**

Peripapillary hyperreflective ovoid mass-like structures (PHOMS) and dome-shaped macula (DSM) are 2 optical coherence tomography findings reported in 2018 and 2008, respectively. To date, there have been no ophthalmic case reports of concomitant PHOMS and DSM.

**Patient concerns:**

A 19-year-old woman presented to our clinic with complaints of decreased vision in both eyes.

**Diagnosis:**

The patient was diagnosed with PHOMS and a dome-shaped macula complicated by subretinal fluid in both eyes.

**Interventions:**

A micropulse laser under the guidance of Indocyanine green angiography was applied to the hyperfluorescent areas and drugs to improve retinal microcirculation.

**Outcomes:**

No response to any intervention over the 41 months of follow-up, her visual acuity remained the same, and the subretinal fluid often recurred.

**Lessons:**

PHOMS and DSM are associated with myopia; myopia may be a mediator between PHOMS and DSM. Dome-like structural changes may occur in different parts of the retina (optic disc and macula), caused by asymmetric myopic posterior scleral growth.

## 1 Introduction

Peripapillary hyperreflective ovoid mass-like structures (PHOMS) and dome-shaped macula (DSM) were 2 optical coherence tomography (OCT) findings in 2018 and 2008, respectively.^[1,2]^ PHOMS can be broadly classified as optic disk edema–associated PHOMS, optic disc drusen (ODD)-associated PHOMS, or anomalous disk–associated PHOMS. PHOMSs are seen in many conditions, including papilledema, non-arteritic anterior ischemic optic neuropathy, central retinal vein occlusion, acute demyelinating optic neuritis, ODD, and tilted disks (myopic obliquely inserted disks), and in many cases resolve along with the underlying condition. For DSM, the normal shape of the macula detected on OCT is altered and inward bulging occurs in a dome-shaped formation. Choroidal neovascularization, serous retinal detachment, and retinal pigment epithelial (RPE) detachment are the most common macular complications that can occur during follow-up of dome-shaped macula.^[3–6]^ To date, there have been no ophthalmic reports of concomitant PHOMS and DSM. In this report, we present the case of a young woman with PHOMS and dome-shaped macula in both eyes, complicated by recurrent subretinal fluid in both eyes.

## 2 Case presentation

A 19-year-old woman presented to our clinic with complaints of decreased vision in both eyes. The patient had a history of high myopia. Best-corrected visual acuity was 20/32 in both eyes. Spheric equivalents were−7.50 in the right eye and−6.50 in the left eye, respectively. The intraocular pressure was 17 mm Hg in the right eye and 15 mm Hg in the left eye. Ocular axial lengths were 25.23 mm in the right eye and 25.05 mm in the left eye. The anterior segment was bilaterally normal. Fundoscopy revealed retinal pigment epithelial changes between the macula and optic disc in both eyes; the boundary of the optic disc was blurred, and temporal choroid atrophy and leopard fundus changed (Figs. 1  [A] and 1 [A’]). Visual field were normal in the both eyes, spectral-domain OCT (Spectralis, Heidelberg Engineering, Heidelberg, Germany) revealed bilateral “C” shaped Peripapillary Hyper-reflective Ovoid Mass-like Structure (PHOMS) and horizontal dome-shaped macula complicated by serous subretinal fluid detachment (Figs. 1  [B,C,D,E], and 1[B’,C’,D’,E’]). Subfoveal choroidal thickness was measured with enhanceddepth imaging optical coherence tomography (EDI-OCT) (Spectralis, Heidelberg Engineering, Heidelberg, Germany) and found 59 μm and 61 μm for right and left eye, measurement of the macular bulge height (Dome-shaped) found 230 μm and 306 μm for right and left eye, respectively (Figs. 1  [D], and 1 [D’]). The analysis of three-dimensional OCT reconstructions also provides additional information on the curvature of the optic disc and macular area (Figs. 2 [A,B], and 2 [A‘,B’]). Optical coherence tomography angiography (RTVue-XR; Optvue, Inc.; Fremont, CA, USA) revealed bilateral choroid atrophy from the optic disc to the central macular, and the great vessel layer of the choroid was visible. No abnormal blood flow signals of new vessels were observed in the central macular(Figs. 3 [A,B,C,D], and 3 [A‘,B’,C’,D’]). Indocyanine green angiography and Fluorescein angiography (Spectralis, Heidelberg Engineering, Heidelberg, Germany) demonstrated hyper-fluorescence due to window defect and surrounding irregular hypo-fluorescent areas in both macula (Figs. 3 [E,F] and 3 [E’,F’]). Choroidal mass was ruled out with ultrasonography. Cranial computed tomography and orbital computed tomography were normal. With these findings, the patient was diagnosed with PHOMS and dome-shaped macula complicated by subretinal fluid in the both eyes. Micro-pulse laser under the guidance of Indocyanine green angiography was applied to the hyperfluorescent areas and drugs to improve retinal microcirculation. No response to any of the interventions. Over the 41 months follow-up, her visual acuities remained the same and the subretinal fluid often recurred.

**Figure 1:**
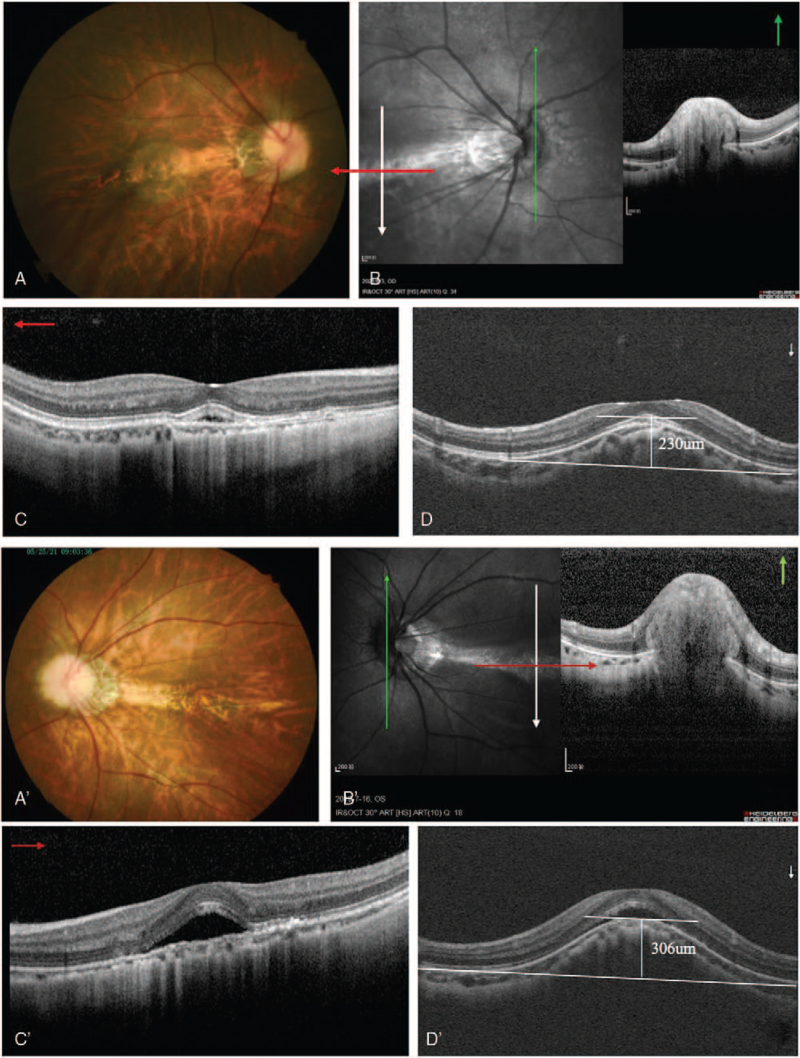
A 1A‘: Color retinography showed retinal pigment epithelial changes between macula and optic disc of both eyes, and the boundary of optic disc was blurred, temporal choroid atrophy, leopard fundus changed. Figures 1 (B,C,D,E) 1(B’,C’,D’,E’): SD-OCT revealed bilateral “C” shaped PHOMS and horizontal dome-shaped macula complicated by serous subretinal fluid detachment. Subfoveal choroidal thickness was measured with EDI-OCT and found 59 μm and 61 μm for right and left eye. Figures 1D, 1D’: measurement of the macular bulge height (Dome-shaped) found 230 μm and 306 μm for right and left eye, respectively. SD-OCT = spectral-domain OCT. PHOMS = peripapillary hyperreflective ovoid mass-like structure EDI-OCT, enhanced depth imaging.

**Figure 1 (Continued):**
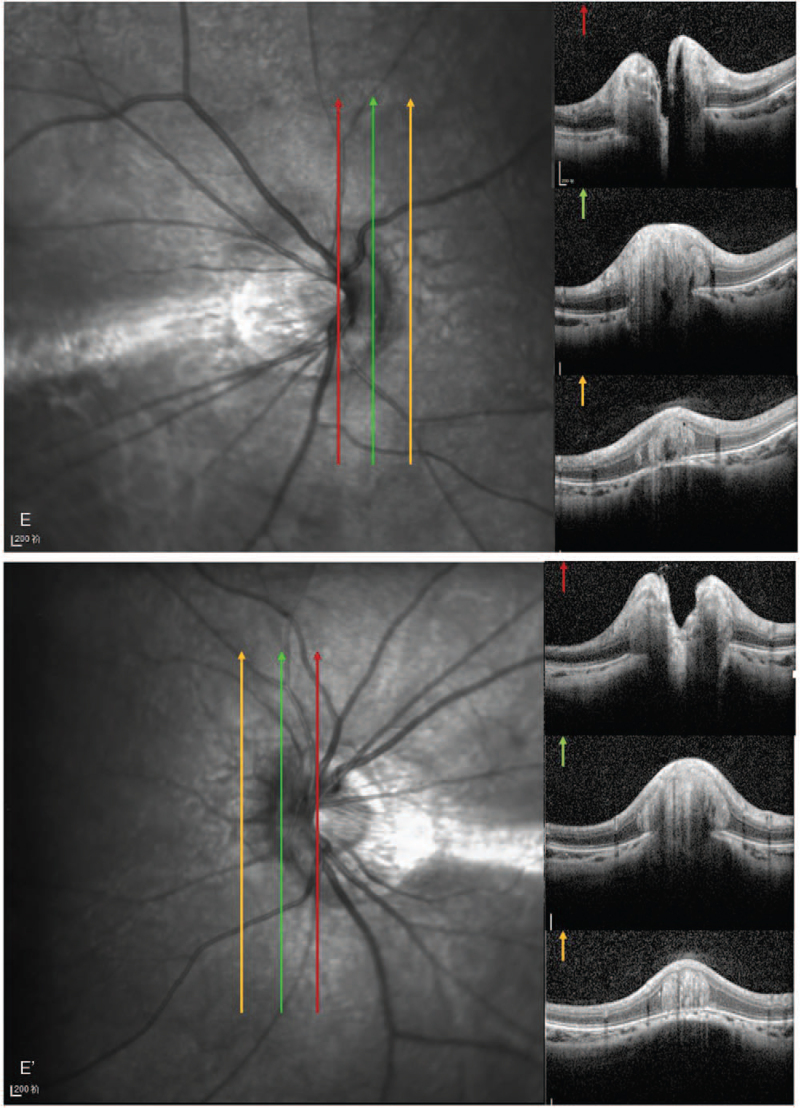
A 1A‘: Color retinography showed retinal pigment epithelial changes between macula and optic disc of both eyes, and the boundary of optic disc was blurred, temporal choroid atrophy, leopard fundus changed. Figures 1 (B,C,D,E) 1(B’,C’,D’,E’): SD-OCT revealed bilateral “C” shaped PHOMS and horizontal dome-shaped macula complicated by serous subretinal fluid detachment. Subfoveal choroidal thickness was measured with EDI-OCT and found 59 μm and 61 μm for right and left eye. Figures 1D, 1D’: measurement of the macular bulge height (Dome-shaped) found 230 μm and 306 μm for right and left eye, respectively. SD-OCT = spectral-domain OCT. PHOMS = peripapillary hyperreflective ovoid mass-like structure EDI-OCT, enhanced depth imaging.

**Figure 2:**
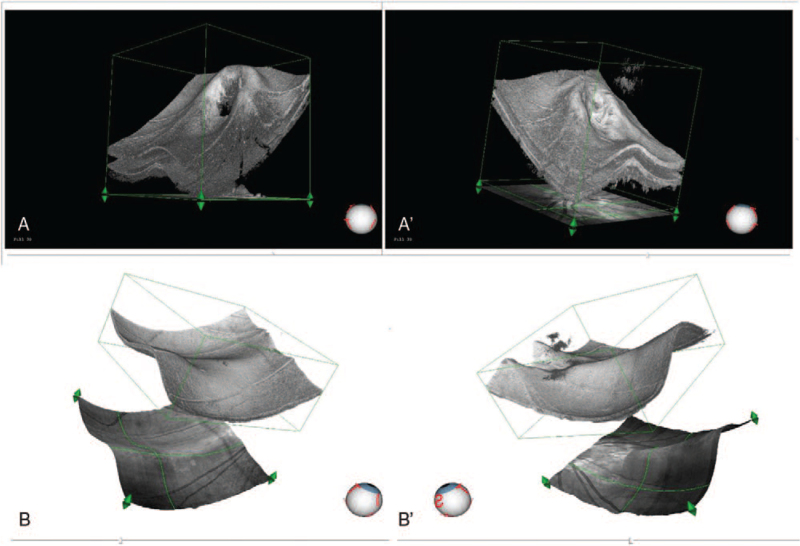
A 2B and Figures 2A‘, B’: The analysis of three-dimensional (3D) OCT reconstructions also provides additional information on the curvature of the optic disc and macular area. 3D-OCT = three-dimensional OCT.

**Figure 3:**
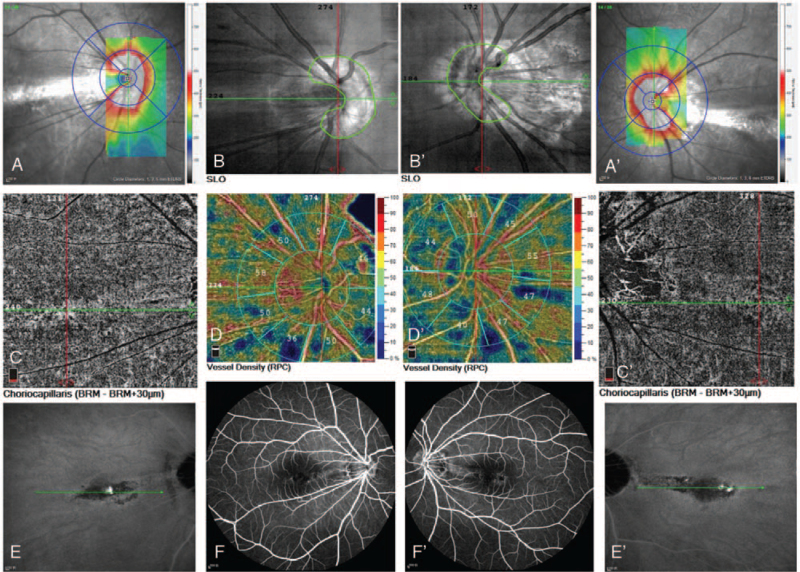
(A,B,C,D) and 3(A‘,B’,C’,D’): OCT-A revealed bilateral choroid atrophy from the optic disc to the central macular, and the great vessel layer of the choroid was visible. No abnormal blood flow signals in new vessels were observed in the central macula. Figures 3 (E, F) and 3 (E‘, F’): ICG-A and FAG demonstrated hyperfluorescence due to window defects and surrounding irregular hypo-fluorescent areas in both maculae. OCT-A, optical coherence tomography angiography ICG-A, indocyanine green angiography FAG, fluorescein angiography.

## 3 Discussion

PHOMS and DSM are new OCT finding.^[1,2]^ The PHOMS is located next to the disk and above the Bruch membrane at the opening of the Bruch membrane opening with a mass-like structure. It is ovoid, and its reflected signal is similar to that of the retinal nerve fiber layer and neural cell layer. A PHOMS in three dimensions is better thought of as a partial torus than a discrete lump—more inner tube than basketball.^[7]^ PHOMS were first observed in the early spectral-domain OCT era, during studies of optic disc swelling and pediatric tilted disc syndrome, in which they were described as “dome-like hyper reflective structures.”^[8]^ They have subsequently been observed in numerous contexts, including papilledema, ODD, nonarteritic anterior ischemic optic neuropathy, central retinal vein occlusion, optic neuritis, and myopic/tilted optic discs.^[9]^ The pathogenesis of PHOMS is suspected to involve herniation of distended axons into the peripapillary retina. Axonal stasis is suggested to be a pathophysiological cause of PHOMS; however, its exact pathophysiology remains unknown. Further research is needed regarding the pathophysiological mechanisms of PHOMS. After ruling out the possibility of PHOMS from other causes, our case of PHOMS may be associated with high myopia and posterior scleral asymmetric growth caused by optic disc dome-like changes (Fig. 2A, 2A’). The treatment of PHOMS is mainly aimed at the primary disease, looking for the primary factors leading to axonal stasis, treating the primary disease, and preventing progressive damage of retinal ganglion cells and retinal nerve fiber layer in time. Therefore, we closely observed patients with high myopia progression rates and timely intervention measures.

DSM was first described by Gaucher et al in eyes with high myopia, an anterior convex protrusion of the macula towards the vitreous cavity observable on OCT.^[2]^ This seems to be related to a localized scleral thickness, which might be the result of regional variation in the scleral biomechanical properties and the process of emmetropization, causing asymmetric scleral growth. The presence of DSM is associated with an increased risk of complications. The clinical spectrum ranges from asymptomatic to metamorphopsia and mild-to-moderate gradual visual loss. Visual impairment in DSM results from retinal pigment epithelial changes, subfoveal serous detachment, retinoschisis, and myopic choroidal neovascularization (CNV).^[3]^ A wide range of therapies have been used to treat subretinal fluid and CNV associated with DSM, including oral eplerenone or acetazolamide, focal laser, intravitreal injections of triamcinolone and anti-vascular endothelial growth factor agents, and half-fluence/half-dose photodynamic therapy; however, these have not been shown to be effective in achieving a significant anatomic or functional response.^[10]^ In our case, horizontal DSM related to high myopia, recurrent subretinal fluid secondary to DSM, DSM had no response to micro-pulse laser and drugs to improve retinal microcirculation, and visual function was not significantly decreased, so it is important to observe the development rate of high myopia and the occurrence of CNV.

## 4 Conclusion

In summary, PHOMS and DSM are associated with myopia, and myopia may be a mediator between PHOMS and DSM. Dome-like structural changes may occur in different parts of the retina (optic disc and macula), caused by asymmetric myopic posterior scleral growth. Attention should be paid to monitoring possible decreased visual function due to progressive damage to the RPE complex. The treatment of PHOMS is mainly aimed at the primary diseases. When DSM occurs in the subretinal fluid and CNV, timely anti-Vascular Endothelial Growth Factor therapy and treatment of the RPE barrier

## Author contributions

**Data curation:** Da-Guang Bi.

**Resources:** Wei Gu.

**Supervision:** Xiao-Yan Peng.

**Writing – original draft:** Wei Zhang.
